# The Uses and Experiences of Synchronous Communication Technology for Home-Dwelling Older Adults in a Home Care Services Context: Qualitative Systematic Review

**DOI:** 10.2196/59285

**Published:** 2024-11-22

**Authors:** Martin Vinther Bavngaard, Anne Lund, Björg Thordardottir, Erik Børve Rasmussen

**Affiliations:** 1 Department of Rehabilitation Science and Health Technology Faculty of Health Sciences Oslo Metropolitan University Oslo Norway; 2 Faculty of Occupational Therapy School of Health, Business and Natural Sciences University of Akureyri Akureyri Iceland; 3 Department of Social Work, Child Welfare and Social Policy Faculty of Social Sciences Oslo Metropolitan University Oslo Norway

**Keywords:** systematic review, qualitative, thematic synthesis, communication technology, relatives, home care services, aging in place, home-dwelling, older adult, aging, gerontology, European, effectiveness, information, technology, health care provider, cross-disciplinary, telehealth, telemonitoring

## Abstract

**Background:**

European health care systems regard information and communication technology as a necessity in supporting future health care provision by community home care services to home-dwelling older adults. Communication technology enabling synchronous communication between 2 or more human actors at a distance constitutes a significant component of this ambition, but few reviews have synthesized research relating to this particular type of technology. As evaluations of information and communication technology in health care services favor measurements of effectiveness over the experiences and dynamics of putting these technologies into use, the nuances involved in technology implementation processes are often omitted.

**Objective:**

This review aims to systematically identify and synthesize qualitative findings on the uses and experiences of synchronous communication technology for home-dwelling older adults in a home care services context.

**Methods:**

The review follows the PRISMA (Preferred Reporting Items for Systematic Reviews and Meta-Analyses) 2020 checklist for reporting. We conducted a cross-disciplinary search in 5 databases for papers published between 2011 and 2023 that yielded 4210 citations. A total of 13 studies were included after 4 screening phases and a subsequent appraisal of methodological quality guided by the Critical Appraisal Skills Programme tool. From these, prespecified data were extracted and incorporated in a 3-stage thematic synthesis producing 4 analytical themes.

**Results:**

The first theme presented the multiple trajectories that older users’ technology acceptance could take, namely straightforward, gradual, partial, and resistance laden, notwithstanding outright rejection. It also emphasized both instrumental and emotional efforts by the older adults’ relatives in facilitating acceptance. Moving beyond acceptance, the second theme foregrounded the different types of work involved in attempts to integrate the technology by older users, their relatives, and health care providers. Theme 3 highlighted how the older users’ physical and cognitive conditions formed a contextual backdrop challenging this integration work, together with challenges related to spatial context. Finally, consequences derived from taking the technology into use could be of a both enabling and complicating nature as integration reconfigured the way users related to themselves and each other.

**Conclusions:**

The acceptance and integration of synchronous communication technology for older adults involves multiple user groups in work tending to the technology, to the users themselves, and to each other through intergroup negotiations. This review’s original contribution consists of its attention to the dynamics across different user groups in deriving consequences from using the technology in question, in addition to its assertion that such consequences may be both intentional and unintentional. We argue that our findings may be used to provide nuance to policies addressing—and practices taking place in—contexts that involve similar user technology constellations to the ones explored in this paper.

**Trial Registration:**

PROSPERO CRD42023414243; https://tinyurl.com/wrha6j3f

## Introduction

### Background

In coming years, the health care needs of increasingly aging populations are projected to supersede the available resources of health care systems in Western societies [1]. Measures have been taken to meet this challenge, with health and social policies across the United Nations prioritizing older adults’ ability to “age in place” [2]—a scenario wished for by most older adults [3]. In several countries, especially established welfare states, the home care services sector has emerged as a political focal point, being first in line to adapt their service provision to the increasing number of home-dwelling older adults [4,5]. Concurrently, Western health policy discourses addressing aging in place construe innovative digital solutions for the care of older adults as enablers per default [6]. Such technologies have been associated with a triple-win narrative: benefitting older adults, the national economy, and society overall [7]. As reflected in the 2020 UN Plan of Action [8], which addresses health and care services globally, emphases on bolstering community home care services coexist with the increased and persistent use of digital solutions. Indeed, research comparing the needs of European home care systems reveals a shared ambition across countries to integrate technology in the provision of home care services [9]. Moreover, existing research has foregrounded the potential held by technology for digital patient service communication in heightening the efficiency of services and enabling health care provision to older adults living in rural areas [10].

However, assessing the impacts of using information and communications technology (ICT) for aging in place requires a wide array of research methodologies and data [11]. Evaluating ICT using only parameters of effectiveness and efficiency runs the risk of presenting a polished but distorted picture, overlooking the dynamics and contextual situatedness of human practices involving technology [12,13]. Even randomized controlled trial designs, the purported “gold standard” of evidence-based health research, have been criticized for being unable to “tell us all we need to know where the intervention is in a complex, dynamic context such as homecare for older people” [14]. As contemporary health and care policies continually favor increased technologization, qualitative knowledge developed from those receiving, using, and experiencing such technology firsthand is of utmost importance [15]. However, this recognition of complex dynamics must also extend to technology. From an interaction design perspective, different technologies *afford* different interactions that influence the dynamics of the user technology interplay [16]. A glance toward the philosophical underpinnings of media theory serves as a further reminder to foreground the medium, that is, technology—through which human interactions transpire [17]. An expanding corpus of qualitative research investigating the integration of ICT by older adults attests to this: while personal alarms may conflict with older adults’ self-identification as independent individuals [18], digital medicine dispensers may cause frustration by imposing rigid structures for medicine taking or by simply not going with the decor [19]. The need to remain attentive to technology types is underlined by Lindberg et al [20], whose systematic review reveals that the tendency to use varying terminologies to describe ICT in home care research is widespread. Indeed, attempts at systematically reviewing existing research on ICT for older adults in the context of home care have conceptualized ICT with varying degrees of specificity, from using umbrella terms, such as “eHealth” [21], “telemedicine” [22], “telecare” [12], “long-distance caregiving technology” [23], and “digital assistive technology” [24], to making more homogenous technology classifications, such as “active ICT” [25] and “apps” [26]. In light of this terminological multiplicity, this review pursues specificity by centering on synchronous communication technology (SCT).

### Review Aim

This systematic review aimed to identify, review, and synthesize results from qualitative studies reporting on the uses and experiences of SCT for home-dwelling older adults in a home care services context. The review operates with the following research question: How is SCT for home-dwelling older adults in a home care services context used and experienced? We also used the following subquestions:

1. What are the types and purposes of SCT investigated in community home care services for home-dwelling older adults?
2. How is the SCT integrated by the users?
3. How is the SCT experienced by the users?

## Methods

### Search Strategy

A protocol detailing the study aim, inclusion criteria, method of extraction, and analysis was registered in PROSPERO before conducting the search [27]. Following preliminary searches used to select and assess the appropriateness of search terms, a systematic search was conducted by the main reviewer (MVB) on March 10, 2023, in the following databases: Web of Science, MEDLINE, PsycINFO, CINAHL, and ACM Digital Library. The search queries used the Boolean phrase search mode and consisted of 4 keyword search strings as well as database-specific index terms (see Table 1 for a detailed overview).

**Table 1:** Search query used in this review^a^ (n=4210).

Database	Results, n (%)
Web of Science	704 (16.72)
MEDLINE	934 (22.19)
PsycINFO	865 (20.55)
CINAHL	568 (13.49)
ACM Digital Library	1139 (27.05)

^a^The following constitutes the 4 core search strings separated by semicolons: (ICT OR “information and communication* technolog*” OR telecare OR ehealth OR “welfare technolog*” OR “warm technolog*” OR “assistive technolog*” OR “care technolog*” OR “social technolog*” OR “communication technolog*” OR gerontechnolog* OR “digital technolog*” OR “digital health” OR virtual OR video OR tablet OR web* OR smartphone* OR “smart device” OR phone* OR mobile* OR computer OR app OR apps OR “mobile application” OR robot* OR platform); (old* OR “old* people” OR “old* adults” OR elder* OR aging OR aging OR senior* OR “later life” OR aged); (“home care” OR homecare OR “elder care” OR “eldercare” OR “home nurs*” OR “domestic care” OR “home based care” OR “home health care” OR “home healthcare” OR “homebased care” OR “community care” OR “community health care” OR “community healthcare”); (qualitative OR experienc* OR perception* OR perceiv* OR feel* OR attitude* OR adopt* OR thematic OR theme* OR ethnograph* OR interview* OR observation* OR phenomenolog* OR “case stud*” OR “focus group” OR narrative* OR “grounded theory”).

### Inclusion Criteria

Because of its frequent use in structuring the research questions of systematic reviews, the qualitative adaptation of the Participants, Interest, and Context model [28] was deemed a suitable tool to trace the review’s area of interest.

#### Participants

This review considered studies involving users of SCT for older adults aged ≥65 years and who are living in their own home. Because of the nature of our phenomenon of interest (see the subsequent section), “users” may refer to participants other than the older adults themselves, such as informal caregivers or health care professionals. No inclusion criteria specified whether the older adults should be living alone or with other people (eg, relatives or spouse) nor if they should be living with or without a chronic condition.

#### Phenomenon of Interest

The review’s phenomenon of interest was uses and experiences with SCT. Drawing on the typology of communication systems in health care by Coiera [29], we define SCT as *a discrete device that enables digital mediation of synchronous communication between 2 or more human actors*. This definition did not limit the technology to a specific purpose (eg, SCT for enhancing social connectedness), allowing us to include a variety of potential purposes while establishing a definitional coherence between the types of technology. As evident in Table 1, the delineation was not reflected in the search queries but instead functioned as a criterion against which potentially relevant studies were manually screened.

#### Context

Only studies investigating the uses and experiences of SCT in home care services were considered for inclusion. Other contexts, for example, hospitals and specialist health care, were excluded. Moreover, this review focused exclusively on original empirical research that was either fully qualitative or with a qualitative component. No other limitations were imposed regarding the study design. Only peer-reviewed publications written in English and published between January 1, 2011, and March 10, 2023, were considered for inclusion. By delimiting this particular period, the review intends to capture the current practices regarding technology in home care services—a setting that has seen an acceleration in the development of technological solutions during the last decade [30]. As such, synthesizing studies published before 2011 may result in lower transferability to current health care systems [31].

### Data Processing

#### Screening and Inclusion

Using the aforementioned search strategy yielded 4210 citations across the 5 databases. Following the search, all identified citations were imported into the reference management software EndNote 20 [32] where 663 (15.74%) duplicates were removed. The remaining 3547 (84.25%) citations were uploaded to the web-based literature review tool Rayyan—Intelligent Systematic Review developed by Ouzzani et al [33] to facilitate the subsequent title and abstract screening process. This process was undertaken in 2 stages. First, 2344 (N=3547, 66.08%) citations that plainly deviated from the inclusion criteria were excluded by the main reviewer (MVB). Second, 3 reviewers (MVB, AL, and EBR) independently screened the title and abstract of the remaining 1203 (N=3547, 33.91%) citations using a screening tool while being blinded to each other’s decisions. Pilot-testing the screening tool revealed that titles and abstracts rarely specified the technology investigated by the study, which made it difficult to accurately judge the technology’s relevance to our area of interest. As a result, a screening tool operating with broader inclusion criteria regarding technology was developed in accordance with the guidelines of Polanin et al [34]. Following the blinded screening, all cases of conflict (259/1203, 21.52%) were resolved through group discussion while using the screening tool.

A total of 250 (20.78%) out of 1203 citations met the inclusion criteria and were retrieved in full-text form. The full-text assessment comprised 2 stages. Because of the impossibility of applying our inclusion criterion pertaining to SCT in the title and abstract screening process, the first stage involved assessing the full-text specifically against this criterion. This assessment was performed by the main reviewer (MVB) and resulted in the exclusion of 193 (N=250, 77.2%) citations. In the second stage, the eligibility criteria were applied to the remaining studies (Textbox 1). Each study was read in full, independently by each member of the review team (MVB, AL, BT, and EBR) and assessed for inclusion. After reaching a consensus through subsequent discussion, a corpus of 18 (N=57, 31%) studies was deemed eligible for inclusion. The screening process was guided by the PRISMA (Preferred Reporting Items for Systematic Reviews and Meta-Analyses) flow diagram [35], modified to reflect our stages of assessment, and is discussed in the Results section.

**Screening tool used for the full-text assessment.**

**Does the study...**

- Investigate the use of “a discrete device that enables digital mediation of synchronous communication between two or more human actors?”
- Relate itself explicitly to older adults?
- Situate itself in a naturalistic home care context?
- Describe its methods as having a qualitative component?

**Is the study...**

- An original primary study (ie, not proceeding or book chapter)?
- Peer reviewed?
- In English?
- Published on or after January 1, 2011?

#### Appraisal of Methodological Quality

Because of the divergent traditions within qualitative research, an awareness of the ways in which studies construct knowledge was necessary [36]. Three reviewers (AL, BT, and EBR) each appraised one-third of the included studies, while MVB appraised all 18 studies using the expanded version of the Critical Appraisal Skills Programme tool [37] by Long et al [38]. As the parameters used to judge methodological quality may vary because of a multiplicity of factors not reflecting actual scientific rigor (eg, reporting style or journal preferences), the appraisal did not rank studies based on their quality nor was a risk of study bias assessment conducted. Consensus regarding all exclusions was reached through discussion. Multimedia Appendix 1 [39-51] presents an overview of the team’s assessments.

### Data Extraction

Data were extracted by the main reviewer (MVB) and cross-checked by coreviewers (AL, BT, and EBR) using a data extraction tool specifically designed for this review. This tool follows an inclusive approach to data extraction [52] by extracting both the “abstract” and “findings” sections as well as the study’s bibliographical and methodological details for conveying its general characteristics in a tabular format.

### Data Synthesis

To ensure transparency, the review was guided by the PRISMA 2020 checklist (Multimedia Appendix 2) for reporting [35]. However, abiding by the qualitative nature of the study aims means casting scientific studies as data themselves. Such studies not only have been developed within a specific social, cultural, and historical context but also present empirical data that have been selected, interpreted, and curated by the respective authors [53]. Therefore, attentiveness toward the constructed nature of “findings” in studies was crucial in establishing the point of departure for—and trajectory of—the synthesis. To preserve an awareness of the context-sensitive nature of each study, its full-text was imported into the qualitative data analysis software, NVivo 12 (Lumivero). However, only the findings were subjected to line-by-line coding.

The findings of the included studies were synthesized using thematic synthesis as described by Thomas and Harden [52]. By anchoring the analysis thematically, the method allows for the synthesization of data developed across different qualitative traditions. Derived from meta-ethnography, thematic synthesis involves a 3-stage process with each stage following an ascending order of analytic abstraction, from (1) coding the extracted data line-by-line to (2) the generation of descriptive and (3) analytic themes. Initial coding was performed independently by MVB and EBR, resulting in 45 and 129 codes, respectively. Although coding many of the same tendencies, MVB’s 45 codes were more developed and thus closer to descriptive themes while EBR’s 129 codes reflected each isolated instance of analytical interest. Incidentally, this allowed for the development of descriptive themes from EBR’s codes while cross-checking these with MVB’s codes—an inductive-deductive pendulation. Where codes did not fit with any descriptive theme, either a new theme was created or the code was deleted. Through this process, 25 descriptive themes were developed. MVB and EBR then proceeded to group the descriptive themes within themes of higher analytical abstraction. In this process, and informed by the account of theme development by Braun and Clarke [54], descriptive themes were dissolved and their codes were distributed into other themes where they proved to be a better fit for the story conveyed by the analytical themes. During this distribution, MVB and EBR checked each code’s consistency with its new placement by revisiting its original context. The total amount of descriptive themes consequently decreased to 21, making up 4 analytical themes. Provided with a mapping of analytical and descriptive themes, AL and BT read through all included studies to assess MVB’s and EBR’s synthesization. A subsequent discussion meeting was held in which the thematization and theme labels were discussed, refined, and agreed upon.

### Ethical Considerations

As the data corpus consisted exclusively of research studies published in peer-reviewed scientific journals, and thus did not entail data collection involving human participants, ethics approval was not sought as part of conducting this review. The ethical integrity of the included studies was assessed as part of the methodological appraisal.

## Results

### Characteristics of the Included Studies

**Figure 1:**
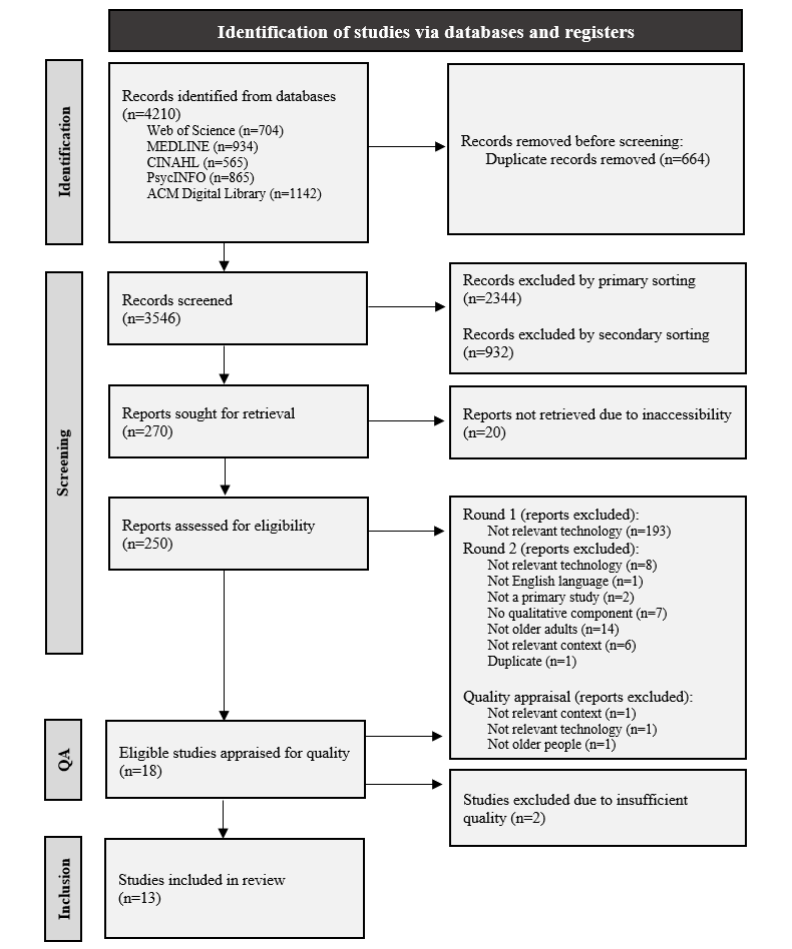
PRISMA (Preferred Reporting Items for Systematic Reviews and Meta-Analyses) flowchart illustrating the process of identifying and screening studies for inclusion. QA: quality appraisal.

The screening process, illustrated using the PRISMA flow diagram in Figure 1, produced 18 studies eligible for inclusion. However, during the methodological appraisal, 2 (N=18, 11%) studies were deemed to be of insufficient quality for the review and 3 (16%) studies did not comply with the inclusion criteria. Hence, they were excluded (Multimedia Appendix 3). Following their removal, the final dataset consisted of 13 studies whose characteristics are outlined in Table 2. The included studies were undertaken in Australia, Canada, Denmark, Finland, Germany, Norway, Spain, Sweden, and the United States and involved a total of 270 participants. Older adults were key participants in all studies. In addition, the studies included informal caregivers; formal carers (nurses, occupational therapists, etc); assessors; interpreters; managers; and palliative care consultation workers. In total, 10 (N=13, 76%) studies collected data from different user groups, although 2 (15%) studies did not disclose the number of participants in each user group. Furthermore, 1 (7%) study specified the different user groups but omitted the number of participants from each. The following technologies were investigated by the studies: alarm pendant (5/13, 38%), tablet with videoconferencing software (3/13, 23%), laptop with videoconferencing software (2/13, 15%), picturephone (1/13, 7%), telephone (1/13, 7%), and video phone (1/13, 7%). The duration of use ranged from participants having used the technology once or twice to ≥12 months. The studies’ designs were either qualitative (11/13, 84%) or mixed methods (2/13, 15%). However, the quality assessment revealed that details on the theoretical and methodological underpinnings were generally underreported (Multimedia Appendix 1). The data collection methods included qualitative interviews (12/13, 92%), observations (including video recordings; 6/13, 46%), document analysis (2/13, 15%), and focus groups (2/13, 15%). Seven (N=13, 53%) studies used more than one qualitative data collection method. For analyzing the data, studies used thematic analysis (5/13, 38%), qualitative content analysis (3/13, 23%), hermeneutic analysis (2/13, 15%), discourse analysis (1/13, 7%), or conversation analysis (1/13, 7%) or did not report a framework for data analysis (1/13, 7%). Seven (53%) of the 13 studies reported using an explicit theoretical framework to guide the data analysis.

**Table 2:** General characteristics of included studies.

Study	Country of study	Aim of study	Context of study	Research design	Participants	Type of technology (discrete device)	Type of technology (device-in-network)	Duration of use	Data types	Data analysis methods	Theoretical framework
Correa and Domènech [39], 2013	Catalonia, Spain	Examine the role of technology in the familial care networks of older adults	Home-based telecare	Qualitative	57 (older adults, informal carers, and formal carers)	Alarm pendant	A monitoring system that involves a domestic terminal and pendant through which the user can communicate with a call center manned by teleoperators.	At least 12 months	Interviews and focus groups	Thematic analysis	Actor-Network Theory
Criado and Domènech [40], 2015	Spain	Explore the impact of home telecare services on the care relations for older adults	Home-based telecare	Qualitative	Not reported (older adults, relatives, and technicians)	Social alarm pendant	An alarm hub and social alarm pendant through which the user can communicate with a call center manned by teleoperators.	Not reported	Interviews and observations	Thematic analysis	Science and technology studies
Gilbert et al [41], 2021	Australia	Explore stakeholders’ perspectives on using video remote interpreting for home-based cognitive assessments	Home-based cognitive assessment	Qualitative	25 (6 older adults, 9 assessors, and 10 interpreters)	Tablet with videoconferencing software	An iPad or Microsoft Surface Pro tablet placed in the homes of users, connecting them to interpreters via one of 2 videoconferencing platforms set up by agency support staff.	At least once	Interviews	Not reported	—^a^
Ilomäki and Ruusuvuori [42], 2022	Finland	Analyze how video-mediated, nurse-led guiding of medicine taking shapes client autonomy	Home-based telecare service pilot	Qualitative	7 (4 older adults and 3 nurses)	Tablet with videoconferencing software	A tablet with videoconferencing software placed in the home of the user connecting them to home care nurses stationed in a shared office.	At least once	Observations (video recordings)	Conversation analysis	Empirical ethics
Karlsen et al [43], 2017	Norway	Explore how older adults and their family caregivers experience the use of telecare services	Home-based telecare	Qualitative	24 (18 older adults and 7 relatives)	Personal alarm (and other)	A wrist- or neck-worn alarm facilitating 2-way communication, primarily used to connect users to home care services.	At least 5 months	Interviews	Hermeneutic analysis	—
Lee et al [44], 2021	United States	Examine the impact of a telephone reassurance program on older adults	Telephone-based social support	Mixed-methods	17 (older adults)	Telephone	Older adults are assigned older volunteers who initiate calls via a landline or mobile telephone.	12 weeks	Interviews	Thematic analysis	Transactional Model of Stress and Coping
Outila and Kiuru [45], 2021	Finland	Analyze older adults’ use of the picturephone in a home care setting	Home-based telecare	Qualitative	8 (older adults)	Picturephone	A tablet placed in the home of the user, connecting them to family, friends, and health care services as well as providing audiovisual content.	At least once	Interviews and observations	Foucauldian discourse analysis	Actor-Network Theory and Foucauldian concepts
Paul et al [46], 2019	Canada	Understand the experience of using mobile web-based videoconferencing for home-based palliative care of older adults	Home-based palliative telehealth	Mixed-methods	30 (8 older adults, 9 relatives, 5 RPC-CT^b^ members, and 8 nurses)	Laptop with videoconferencing software	A laptop with an external webcam and speakerphone placed in the home of the user with assistance from a nurse, connecting them to a palliative care physician consultant.	Not reported	Interviews and focus groups	Content analysis	—
Schmidt et al [47], 2019	Germany	Understand how the video-mediated monitoring process of care and case management is experienced by older adults	Case and care management	Qualitative	20 (older adults)	Tablet with videoconferencing software	A tablet with videoconferencing software placed in the home of the user, connecting them to nurses, social workers, and peers with similar characteristics.	9 months	Interviews	Interpretative-hermeneutic analysis	—
Similä et al [48], 2014	Finland	Explore the experienced value of a video-mediated service by health care professionals and older users	Tele-delivered rehabilitation and occupational therapy	Qualitative	8 (5 older adults and 3 occupational therapists)	Laptop with videoconferencing software or broadcasting device	A laptop with videoconferencing software and an external webcam and speakers or Vidyo HD-50 videoconferencing attachment with touch computers for input.	10 months	Interviews, observations, and documents	Content analysis	—
Stokke [49], 2017	Norway	Understand the microcontext of the coproduction of care involving technologies used in care practices	Home-based telecare	Qualitative	24 (11 older adults, 4 relatives, 6 care workers, and 3 key workers or managers)	Social alarm pendant	A unit placed in the home of the user, encompassing a wrist- or neck-worn device connecting the user to a call center responder.	At least 12 months	Interviews and observations	Thematic analysis	Script theory and Domestication theory
Stokke [50], 2017	Norway	Explore how actors using a social alarm relate to, perceive, and articulate their expectations of the technology in everyday life	Home-based telecare	Qualitative	30 (11 older adults, 9 relatives, 7 care workers, and 3 key workers or managers)	Social alarm pendant	A unit placed in the home of the user, encompassing a wrist- or neck-worn device connecting the user to a call center responder.	At least 12 months	Interviews, observations, and documents	Thematic analysis	Script theory and Domestication theory
Åkerlind et al [51], 2018	Sweden	Describe how older adults with e–home care and their relatives understand and experience safety in everyday life	Home-based telecare	Qualitative	20 (12 older adults and 8 relatives)	Video phone (and other)	A video screen–based device placed in the home of the user, connecting to the computer of e–home care staff or relatives and offering video calls.	6 months	Interviews	Content analysis	—

^a^Not applicable.

^b^RPC-CT: rural palliative care consultation team.

### Synthesized Findings: Analytical Themes

#### Overview of Themes

Four analytical themes were developed, forming a synthesized narrative depicting the multifaceted process of accepting and integrating the technology along with factors moderating this process and the various consequences of use. Through this narrative, the synthesis addresses the uses and experiences of SCT for home-dwelling older adults. The four themes are interconnected and sequentially ordered as follows: (1) *crossing the doorstep: trajectories toward technology integration*; (2) *making it fit: types of integration work*; (3) *complicated by context: physical, cognitive, and spatial challenges*; and (4) *putting it to use: (un)intended consequences of using the technology*. For the finalized thematic hierarchy, see Textbox 2.

**Depiction of the synthesis’ thematic hierarchy, including themes and subthemes.**

**Crossing the doorstep: trajectories toward technology integration**

- Antecedent perceptions of the technology
- Acceptance
- Temporal acceptance
- Partial acceptance
- Resistance

**Making it fit: types of integration work**

- Older user’s work
- Caregiver work
- Service work
- Shifting roles
- Cultural and technological role expectations

**Complicated by context: physical, cognitive, and spatial challenges**

- Physical issues
- Significance of spatial context
- Cognitive issues

**Putting it to use: (un)intended consequences of using the technology**

- Technology as enabling interaction
- Technology as complicating interaction
- Technology as structuring interaction
- Social connectedness
- Creating new situations with uncertain norms
- Safety and security
- Worries
- Relief

#### Theme 1: Crossing the Doorstep—Trajectories Toward Technology Integration

A recurring topic across the included studies was the initial acceptance or rejection of the technology by the end users, primarily the home-dwelling older adults. As indicated by our sequential ordering of the 4 themes, acceptance is understood here as a precursor to the work involved with integrating the technology into the lives of its users. The present theme, then, centers around the phase of accepting the technology, thereby beginning the process of turning oneself from a nonuser to a user by integrating or “taming” it [55]. To expand upon the dynamics of the theme, we offer the metaphor of the user standing opposite the technology in a doorway.

While all studies reported on cases where the technology was welcomed across the doorstep more or less straightforwardly by older users, the synthesis also showed a variety of other responses. One such response was *gradual* acceptance—the technology was admitted, but with initial suspicion. Illustrating this, Stokke [49] sums up the journey of one older user initially rejecting the technology but later welcoming it: “He needed time to get used to it before accepting it.” Full acceptance was achieved over time, conditional on the older user being familiarized with, and learning how to use, the technology [45,48-50].

Another response landed acceptance at a less stable form, namely one of *partiality*. In such cases, the user left the door ajar—not fully welcoming the technology but not rejecting it either. Users did so for different reasons, one being the sense that use involved compromise. Some older users of social alarms felt that use entailed a compromise with their self-perception by accepting their fragility [39,50]. Another reason was that the technology did not live up to their expectations, expressed as users’ discontent with the current functions of their picturephone [45]. Similarly, cost also spurred hesitation among some older users [45]. In cases where health care professionals constituted end users, partial acceptance arose from views that video communication technology compromised quality service provision when compared with face-to-face interaction [41,44-46,48].

A final type of response involved attempts by older users to *reject* the technology. Reasons for rejection were dissatisfaction with the technology’s qualities, requirements of constant tinkering to ensure functionality, convictions that engaging with the technology was not worth the effort, and unwillingness to pay money for the technology as part of health care services [45,48]. Some attempts at keeping the technology out of the household were successful, as shown in a case description by Criado and Domènech [40]. Here, a potential older user refused to allow a telecare installer to put up the telecare device that her daughter had suggested, instead escorting the installer out of her home. However, not all acts of resistance resulted in outright rejection. Some older users chose simply to ignore the technology in question [45] or to resist through intentional nonuse, as disclosed by one older user of a social alarm pendant: “In the beginning, I must say...I thought it was disgusting to wear...I did not like it. I took it off at night and put it on the bedside table” [50]. Although the technology had slipped past the doorstep, it was treated with derision as an unwelcome guest.

Across the various types of responses, relatives and informal caregivers were commonly reported as being *driving forces of acceptance* [40,43,45,50]. Whether gradual, partial, or more straightforward, acceptance was thus often found to be heavily influenced by efforts from relatives. These efforts were both instrumental, for example, by suggesting and acquiring the technology or directing the older user on how to use it, and emotional, for example, by convincing the older adult to accept the technology (eg, by framing the introduction of technology into their lives as an inevitability) [40,45]. However, the involvement of relatives could also create tensions between them and the older users. For instance, when relatives were interested in the technology but the older users were not [40,43]. One study [39] poignantly illustrates such a scenario through excerpts of a focus group interview with telecare personnel:

[W]hen a son or daughter requests it, which happens a lot.... The person who has to want it is them, because if you want it we’re going to set it up and they’re going to say, “Yes, yes, yes,” so we shut up, so that we leave them alone, and when we leave they’re going to take it off or leave it on top of the nightstand.

As indicated by the excerpt, initial acceptance was sometimes provided by relatives rather than the older adults themselves because of concerns regarding their safety [39,40,43,50].

Finally, another driving force was older adults’ existing perceptions, either of the technologies in question or of new technology altogether. Continuing the doorway metaphor, such perceptions are perhaps like judging the merits of who is at the door through the peephole. In some cases, existing perceptions were a driving force of rejection. For instance, the peephole glance led to skepticism of the picturephone even before it was taken into use [45]. Similarly, expecting to fail or becoming confused deterred some older adults from initially accepting videoconferencing [48]. However, we stress that the studies also found the opposite, namely older adults who viewed the prospect of new technology with curiosity [45] or as a helpful addition for managing future health-related needs [43,45]. Schmidt et al [47] noted that *all* older users had positive expectations for the videoconferencing technology.

#### Theme 2: Making It Fit—Types of Integration Work

Having accepted the technology, although sometimes only partially, users began the process of integrating the technology into their respective contexts. This theme illustrates how attempts at integration induce change and foregrounds the types of work performed by different users to manage these changes related to the process of integration. The theme also encompasses roles and cultural expectations, as each manifest through the integration work done by users.

Principally, *older users* engaged in work aimed at making the technology fit into their everyday life. For some, this meant not only moving furniture to accommodate the technology [45] but also learning how to use the technology, for instance, by reorganizing existing schedules or appropriating their technology use to align with their individual interests [40,45]. Several older users were enrolled in work tending to the technology by ensuring that power and internet cables were plugged in [48] and that the technology was working properly [39]. In assessing whether her home terminal was functional, one relative recounted this latter type of work in which an older user of a social alarm accepted her role as guarantor [39]:

[S]he says: “I got a call to check on it. I got a call from the alarm center to check to see whether...” Well, you know, if the device is working. They tell her what to press and then that’s all. So she tells us about it if we are not at home.

The work of integrating the technology also invoked *relatives*, often in the role of informal caregivers, to support the use of the technology by the older user on an instrumental level [39]. Such work included instructing the older user to remember to use the technology and continually suggesting use purposes [43]. Some studies found that relatives’ work was crucial for sustained use of the technology [39,43,45], with Outila and Kiuru [45] highlighting the breakdown of integration caused by a relative unwilling to support its use:

Interviewer: Well, have you talked to anyone about the possibility of contacting your loved ones via picturephone? Have you had any discussions about that?

Helena: No, I haven’t, not yet. There is, in that paper, [instructions] saying that you can. But my son, he is living his own life, so he doesn’t really care about my things.

Most of the studies described work performed by *service providers* in integrating the technology. First, work was necessary to adapt to the technology-induced changes in service provision. Health care professionals had to negotiate what constituted the appropriate use of videoconferencing technology when assessing older users [46], how to distribute knowledge between teams when conducting video-based cognitive assessments [41], and how to transform content into digital formats [48]. Second, health care services operating the technology needed to allot time and resources to sustain normal operation. Such work included providing adequate technical support for videoconferencing, allocating more time for video-based health care service provision vis-á-vis usual formats, instructing end users on how to use the technology, and conducting routine checks of social alarms’ functionality [39,40,46,48,50].

Furthermore, some integration work enrolled multiple user groups: relatives navigated areas of responsibilities and acted as mediating links between user and home care services [40,43] and services worked to negotiate appropriate contexts for using technology for themselves, older users, and their relatives [50]. During these processes, their work intertwined. Indeed, 4 (80%) of the 5 studies investigating social alarm pendants reported shifts in user roles and role relations among older users, relatives, and health care services. Existing relationships between older users and relatives were destabilized by changes brought about by the technology (or its process of installation), sometimes offering opportunities to renegotiate forms of relatedness [39,40]. However, reports also indicate that precisely because of technology-induced changes to existing roles and role relations, disputes over care responsibilities may arise. Relatives involved in work to ensure the social alarms’ functionality may acquire technological expertise surpassing that of health care professionals [43]. Similarly, ensuring the optimal function of the social alarms in some cases meant diverging from care constellations preferred by the older user or meant that relatives otherwise caring for the older user did not fit the role prescribed by social alarms [40].

This notion of roles points to a more general finding synthesized across several studies—that when the technology was integrated into the lives of its users, norms and expectations surfaced, pointing to underlying culturally contingent ideal role figurations between older users, relatives, and health care services [40,43,45,51]. The issue of “role fit” [40] presented earlier indicates how services operating the social alarm inscribed expectations into the device regarding who could and could not fit the role of carer-through-telecare—and how this clashed with cultural expectations of family members as carers. A daughter’s comments on watching over her mother using a videophone constitute another example [51]. She explains that she views checking up digitally as a “part of everyday modern life” due to emerging possibilities for monitoring using technological devices. These findings foreground that roles involved in technology integration are not just instantiated based solely on functionality. Rather, roles and role figurations draw on preexisting belief systems grounded in cultural understandings, of both care and technologies.

#### Theme 3: Complicated by Context—Physical, Cognitive, and Spatial Challenges

As illustrated by the second theme, integrating the technology entail multiple practices. These practices were sometimes complicated by the presence of challenges pertaining to material and health-related circumstances. Only 3 (N=13, 23%) included studies provided a complete overview of the participants’ health conditions [43,44,48], although several studies reported that health-related conditions of the older users influenced their ability to integrate the technology. Specifically, the presence of *physical or cognitive issues* complicated or hindered integration work altogether. Three (N=13, 23%) studies [41,44,45] recounted users who viewed the requirements for using the technology as insurmountable due to their deteriorating physical health. One older picturephone user described how her disposition changed due to tiredness despite initially accepting the technology: “I was too tired. I was in such a bad condition that I had to let go. And now, for at least three months, I haven’t turned it on at all” [45].

Illustrating practical challenges involving physical health, Gilbert et al [41] note how remote interpreting using videoconferencing software sometimes posed requirements of hearing and vision that the older users could not fulfill. Intertwined with this inability to use the technology was also a decrease in interest to use, although reports omit the specifics of this disinterest. Lee et al [44] highlight a situation in which an older user lost interest during the process of integration as the technology seemed less relevant when viewed from her position of increased vulnerability. Similarly, one older user’s son felt that the technology was not a suitable solution for his mother because of her poor physical condition [51]. Thus, physical health issues were reported to influence both ability and interest to use.

However, most of the reported challenges were related to the older users’ cognitive capacity. Several studies noted how older users lacked the required cognitive capacity to use the technology. For instance, using videoconferencing software demanded concentration and effort beyond that of face-to-face communication to follow the conversation [51] or required too steep of a learning curve. Some users reported that integrating the technology and becoming a “fluent user” [45] was challenged by users’ own perceptions of their capacity to learn at their age. In addition, using the technology could require skills of comprehension not attainable by some older users. According to an occupational therapist, video-mediated therapy requires users to comprehend the norms of video communication, which was not always the case [48]. Similarly, users with cognitive issues sometimes forgot who was on the screen [46]. Stokke [50] vividly depicts how a social alarm, designed to be easy to operate by older adults, nonetheless relies on its user’s ability to understand its function when in need:

Marie was the one wearing the alarm. She was physically vigorous, even though she had had a stroke some years back and suffered from dementia. Marie did not understand how she should call for help when her husband got ill, and Peter was too heavy for Marie to manage.... When he experienced falls in blood pressure, he passed out for a while. Sometimes she pressed the pendant, sometimes not. She got confused when these things happened, but then he woke up and said, “Press the button!”

Paralleling the findings of Stokke [50], some studies included comments made by informal caregivers and health care professionals in which the technology in question was deemed inappropriate to use with or by older users with dementia [41,49]. In these ways and others, the older users’ deteriorating health came in the way of performing the necessary integration work.

Beyond health, issues related to spatial context were also reported to influence integration work. First, such influences related to the spatial arrangement of the technology, where suboptimal lighting conditions [41,46], background noise, and disturbances by other people present [41,48] were noted by health care professionals as consequences of poor placement of the technology in the home of the older user. By the same token, Ilomäki and Ruusuvuori [42] illustrate that guiding medicine taking may be disrupted by too far of a distance between medicine and videoconferencing equipment. Second, several studies detailed how the physical presence or absence of human actors affected the integration of the technology [41,43,46,47]. For example, the presence of relatives during the installation and maintenance of older users’ social alarms was emphasized by Criado and Domènech [40], while Outila and Kiuru [45] stated that a lack of nearby relatives acting as technology aides hindered the use of the picturephone.

#### Theme 4: Putting It to Use—(Un)intended Consequences of Using the Technology

While the 2 preceding themes synthesized findings concerning (challenges to) work involved in integrating the technology into the lives of its users, this theme focuses on the reported consequences that arise from having taken the technology into use across user groups. Depending on their process of integration, users derived a wealth of different experiences from interacting with technology for different purposes.

First, older users expressed how using SCT gave rise to feelings of *social connectedness* with peers or relatives despite being isolated either at home or in the hospital [45,47,48,51] and decreased feelings of loneliness [44,45]. Even routine checks performed by health care services made a social alarm user feel less alone, as commented by one relative [39].

Second, feelings of increased *safety and security* were reported by many users. Older users felt that using the technology allowed a sense of relatives or services to be “distant but present,” which provided them with safety while enabling them to live at home with increased independence [39,43,49,51]. By the same token, relatives connecting to the older user via technology also felt safer [39,43,49,51]. While older users felt both connected and safer, users in health care services felt that video communication enabled effective health care provision at a distance owing to visual access [41,45,50] and made encounters with older users more succinct by sidestepping informal chatter [41] and allowing physicians to interact directly with patients [46]. Only one experience of increased safety was reported by health care services. This was concerned with the ability of videoconferencing technology to mitigate the need for service provision in unfamiliar settings and in the presence of strangers [41].

Related to, but separate from, safety and security were reports that using the technology brought users *relief*. Older users said they experienced relief as the technology reduced personal health care costs by replacing physical visits [45] and as they did not need to involve worried relatives in case of an emergency [39]. Relatives felt that being present despite distance relieved them of some care responsibilities [50], allowing them enough peace of mind to go on vacation or simply sleep at night [39,51]. Using the technology was experienced by services as saving time and costs by circumventing travel [41,46,48] and by ensuring fragile older patients easy access to help if needed [45,50].

Contrasting the preceding points, some users experienced the technology as inducing *worries*. Primarily experienced by older users, worries were related to the use of the technology, revolving around technical malfunctions [39,41,51] and internet security [46]. Some relatives worried that the technology could extend older users’ life at home beyond what they deemed appropriate in terms of safety [43,51]. One relative and older user aired their concern that using technology may lessen human contact in future health care service provision for older adults [45,51].

Far from stable cause-and-effect end points, the aforementioned experiences are consequences resting upon the dynamics of users’ integration work. We now turn to these dynamics, denoting the ways in which the use of technology produced new situations, sometimes creating uncertain norms challenging the users’ actions, habits, and beliefs. Nowhere is this seen clearer than in older users’ negotiation of what constitutes appropriate and inappropriate use [39,43-45,49,50]. Exemplifying this, one older user recounts her choice not to use her social alarm pendant after a fall despite it being intended for emergencies [39]:

[T]he chair fell down over that way and I fell the other way.... “Ok, everything hurts but no, I haven’t broken anything because the fall was soft. No, so get up,” and of course I wasn’t going to call because you’re not going to stir things up just for something like this.

This excerpt illustrates how acquiring and integrating the technology introduces questions of what it means to be in need. Similarly, one older user sometimes felt the need to test his alarm pendant despite it being for emergency use only and navigated this question of legitimate use by using his humoristic nature and well-established relationship with the health care unit [49]. In several studies, it thus became clear that the established figuration surrounding the technology left open a degree of interpretation for its users to negotiate. Correspondingly, variations in implementation and ambivalences regarding the end purpose of the technology arose [43,45,50].

However, the work of integrating the technology also influenced interactions between users by imposing different interactional premises. Numerous studies reported on how such premises complicated interactions for the respective users, although primarily from the perspective of health care professionals. The imposed format of synchronous audiovisual communication gave rise to interactional dysfluencies: turn-taking became more difficult [41,42,48], conversations were halted by audio and video delays or blackouts [41,46,47], and empathetic or gestural displays became restricted [41]. In addition, some health care professionals stated that discussing sensitive topics was unsuitable using the format [41,46]. Interaction could also be complicated by the spatial separation of interlocutors, as it meant that health care professionals relinquished control of the other user’s side. They were unable to gain a sense of the older user’s settings, including who else was present during the conversation [41,46], and found it difficult to direct the older user’s attention back to the conversation at hand if they went off-topic [41,48]. Furthermore, the capacity to connect instantly to older users meant not warming up and losing interprofessional coordination in advance of the encounter, thereby complicating interaction [41].

Finally, establishing social alarm “roles” restructured existing figurations and therefore interactions between older users, their relatives, and the involved health care services in ways that created tension [39]. The following excerpt illustrates this restructuring [50]:

[T]he care-worker was usually helping other patients when they received an alarm. Due to confidentiality, they had to leave the room to talk and were therefore interrupted in their work. There are many field notes describing how the person receiving help commented on this, as he/she had to wait for the care-worker to return and continue the work.

Here, the prioritization of social alarms by the home care service meant that all other care tasks became subject to potential disruption in case an alarm went off. Thus, the technology created more fragile situations in which distant actors were able to interfere with the situation at hand.

## Discussion

### Principal Findings

By identifying, assessing, and synthesizing findings across 13 qualitative studies, this review provides an understanding of the nuances of how SCT for home-dwelling older adults in a home care services context is used and experienced. Using the method proposed by Thomas and Harden [52] for thematic synthesis enabled an *upcycling* [56] of the findings presented by the included primary studies through analytical abstraction, thereby generating a conceptually innovative sequential understanding of technology use. While other reviews share our overall area of interest, they approach this from different perspectives. These either focus exclusively on the experiences of older adults [12,21,57,58] or include other actors but only when the older adults live with dementia [26,31,59]. Few review studies focalizing on SCT exist; only the scoping review by Chelongar and Ajami [25] operates with a similar definition but includes both qualitative and quantitative studies and omits synthesization. Our review’s innovative contribution is its attention to both the uses and experiences of SCT as contingent on users other than the older user, as well as the technology’s imposition of new premises for their interactions and relatedness.

Although the first theme of the synthesis indicates that acceptance, even if partial, takes as its premise the willingness of the older adult to transform from nonuser to user, one central aspect of our results relates to the enrollment of other users in facilitating acceptance by older users. Relatives and—to a lesser extent—service providers often work to acquire the technology and to persuade the older user to use it. Using the term “warm experts,” Olsson and Viscovi [60] emphasize the role of grown children in appointing, acquiring, and supporting the use of technological devices by their parents, drawing on their familiarity with their qualities and needs. This review echoes such findings but also accentuates the sometimes conflicting values and roles between different users—particularly relatives and older adults. These contest the unequivocal *warmth* of relatives’ engagements. When relatives pursue acceptance despite their parents’ unenthusiasm or act as driving forces in initially acquiring the technology, the notion of older users’ acceptance may be construed as a door to be breached rather than to be let through. Although several included studies attest to this inference, a few reported cases juxtaposed the driving forces; for example, a daughter’s compliance with her father’s wish to age at home using technology despite her conviction that a nursing home might be a better option. The emphasis on the older user’s acceptance may rest partly on the review’s focus on SCT, as older users must inhabit a role in 1 of 2 possible ends of the medium. However, the prevalent preoccupation with older adults’ technology acceptance in research [61-63] lends credence to acceptance being a crucial stage for technology integration. Therefore, it is notable that acceptance among relatives and service providers is a matter largely omitted by the included studies. Given their significance as proxies for older adults’ acceptance of SCT, emphasis on these user groups may be productive when investigating SCT for home-dwelling older adults.

The synthesis ventures beyond acceptance, with the second theme bringing different users’ continuous integration work to the fore and highlighting the dynamics of roles, expectations, and the technology’s fit into existing relations. In this regard, it engages with numerous studies operationalizing the concept of *domestication* to investigate the sociotechnical processes of integration mediating technological “effects” [64-67]. In the context of eldercare, Saborowski and Kollak [68] argue that, rather than being of a technical nature, the question of whether telecare technology is “good” hinges on its domestication into the existing care network. Such understandings of technology integration run parallel to calls for more nuanced approaches to the evaluation of technologies in health care [69-71]. Far from a cry from specialized fields within social science, a recent review investigating knowledge gaps in existing research on the implementation of welfare technology reached a similar conclusion—that research should address longitudinal processes; involve multiple users; and include their attitudes, beliefs, and actual uses [72].

By highlighting the weight given to the physical and cognitive capacities of older users by the included studies, the third theme simultaneously reveals that the competencies of other users and their context—be it organizational or familial—received less attention. This finding aligns with the assertion of Peine and Neven [73] that the development of technology intended for use by older people disproportionally casts their disabilities and needs as the primary issue to solve. In contrast, the study by Nordtug et al [74] on video consultations (a technology comparable to that investigated by 6 of the 13, 46% studies) revealed that uncertainties among general practitioners significantly influenced their integration and use. In this regard, the context surrounding technology use should be considered. All the included studies investigated digitally delivered home-based care—a cultural context with certain conventions potentially framing users’ interaction with the technology [16]. The included SCT can be argued to mirror such a context, as it established a sender-recipient relationship paralleling traditional care pathways. The only exception is the picturephone [45]. Interestingly, this is also the only case in which older users complained of device costs, suggesting that the older users inhabited a consumer role in acquiring the picturephone and—by extension—the selection of that particular care pathway.

The significance of context notwithstanding, our findings point to the general consequences of using the technology across the included studies. Older users benefitted from being able to contact relatives and peers, thereby feeling more socially connected and safer despite isolation. Although only Lee et al [44] explicitly sought to investigate the impacts of technologically mediated interactions on loneliness, a total of 6 (N=13, 46%) studies reported increased social connectedness among older users. Such a discrepancy points to the agency of users in defining the meaning of their engagements with SCT irrespective of its originally intended purposes. Another enablement experienced by older users and, principally, service providers was that of avoiding travel. For service providers, this equated to saving resources. The realization of this particular capacity of SCT is in line with the framing of technology implementation in health care in European countries as a cost-effective measure [8].

### Limitations

Qualitative systematic reviews often seek heterogeneity to maximize nuance across included studies [52,75]. In contrast, our review intentionally delineated technologies of interest as those capable of mediating synchronous communication between human actors. On the basis of the assumption that the characteristics of a technology matter more than its status, we sought technological homogeneity to achieve better comparability. This strategy may indeed have resulted in the loss of valuable variations in use cases and experiences. During the database query, our interest in a particular technological type influenced our choice of search terms; we abstained from relying only on umbrella terms (eg, telecare and eHealth) because of their breadth and included specific technologies (eg, tablets and apps). Beyond revealing what technologies we were interested in, this may have skewed the search toward technology types well-known to us. Nonetheless, this delineation proved productive, revealing a potential for future reviews to address other technologies (eg, social robots or electronic medical record systems), while remaining attentive to their specificities.

Choosing the community home care setting as the review’s area of inquiry gives precedence to countries operating with this organizational context of home care, thereby compromising transferability to differently organized systems of home care provision. On a more general note, we advocate for a principle of selective and informed transferability when attempting to apply the findings of this qualitative meta-synthesis—that by affording transparency regarding the review process, we provide readers with the means to make well-informed decisions about the applicability of our results to their own context [75]. Thus, we do not assume our results are transferable to all contexts—not only due to the aforementioned technological and organizational specificities but also due to the nature of qualitative research itself [53]. Similarly, several aspects of our approach compromise the reproducibility of this review. Framing subjectivity as a precondition for interpretative work rather than as a risk factor to be eliminated, diverging perspectives emerging between reviewers during the processes of screening, appraising, and synthesizing, were viewed positively—as a crossroad involving productive negotiations between alternative interpretations. Discussion meetings were held throughout these processes to align perspectives and achieve transparency but without the conviction that standardized procedures will or must produce the same outcome across all reviewers [56].

### Conclusions

Synthesizing findings across 13 studies, our review aimed to enrich knowledge on the uses and experiences of SCT for home-dwelling older adults in a home care context. Four analytical themes composed a sequential, multifaceted process illustrating first the gradual, partial, and resistance-laden dimensions of accepting technology by older users, and the role of relatives as mediators therein. Succeeding acceptance, the integration of the technology implies the introduction of change, demanding that older users, relatives, and health care providers address these by engaging in work and to tend to the technology’s functioning, to its sustained use, and to each other. Contextual issues complicate these practices, a notable concentration of which concern the qualities of older users specifically. Feelings of social connectedness, safety and security, relief, and worries are not just consequences of use but also consequences of profound reconfigurations of practices and relations across different users. To our knowledge, this is the first review to synthesize scientific reports involving this particular constellation of technologies, users, and contexts. On account of this specificity, we argue that our findings may be transferable to policies aimed at—and practices within—similar domains. At the least, it invites a more nuanced understanding of the implications of introducing SCT into the lives of its users.

## Appendix

Multimedia Appendix 1 Decision summary based on the expanded Critical Appraisal Skills Programme tool.

Multimedia Appendix 2 PRISMA (Preferred Reporting Items for Systematic Reviews and Meta-Analyses) 2020 checklist.

Multimedia Appendix 3 Studies excluded because of insufficient methodological quality.

## Abbreviations

ICT: information and communications technology
PRISMA: Preferred Reporting Items for Systematic Reviews and Meta-Analyses
SCT: synchronous communication technology

## References

[1] (2024). Ageing and health. World Health Organization.

[2] (2022). Ageing policy in Europe, North America and Central Asia in 2017-2022. United Nations Economic Commission for Europe.

[3] Global health and aging. National Institute on Ageing, National Institutes of Health, U.S. Department of Health and Human Services.

[4] Communication from the commission to the European Parliament, the European Council, the Council, the European Economic and Social Committee and the Committee of the Regions REPowerEU plan. European Union.

[5] NOU 2023: 4 tid for handling — personellet i en bærekraftig helse- og omsorgstjeneste. Regjeringen.no.

[6] Lindeman DA, Kim KK, Gladstone C, Apesoa-Varano EC (2020). Technology and caregiving: emerging interventions and directions for research. Gerontologist.

[7] Neven L, Peine A (2017). From triple win to triple sin: how a problematic future discourse is shaping the way people age with technology. Societies.

[8] UN decade of healthy ageing: plan of action 2021-2030. World Health Organization.

[9] Hammink C, Mohammadi M (2024). Uncovering homecare needs and challenges across Europe. Interreg ACE Project.

[10] Rush KL, Singh S, Seaton CL, Burton L, Li E, Jones C, Davis JC, Hasan K, Kern B, Janke R (2022). Telehealth use for enhancing the health of rural older adults: a systematic mixed studies review. Gerontologist.

[11] Connelly K, ur Rehman Laghari K, Mokhtari M, Falk TH (2014). Approaches to understanding the impact of technologies for aging in place: a mini-review. Gerontology.

[12] Karlsen C, Ludvigsen MS, Moe CE, Haraldstad K, Thygesen E (2017). Experiences of community-dwelling older adults with the use of telecare in home care services: a qualitative systematic review. JBI Database System Rev Implement Rep.

[13] Lynch J, Hughes G, Papoutsi C, Wherton J, A'Court C (2022). "It's no good but at least I've always got it round my neck": a postphenomenological analysis of reassurance in assistive technology use by older people. Soc Sci Med.

[14] Mort M, Roberts C, Pols J, Domenech M, Moser I, EFORTT investigators (2015). Ethical implications of home telecare for older people: a framework derived from a multisited participative study. Health Expect.

[15] Stokke R, Hellesø R, Sogstad M (2019). Hvorfor er det så vanskelig å integrere velferdsteknologii omsorgstjenesten?. Tidsskrift Omsorgsforskning.

[16] Norman D (2013). The Design of Everyday Things.

[17] McLuhan M (1994). Understanding Media: The Extensions of Man.

[18] Peek ST, Luijkx KG, Rijnaard MD, Nieboer ME, van der Voort CS, Aarts S, van Hoof J, Vrijhoef HJ, Wouters EJ (2016). Older adults' reasons for using technology while aging in place. Gerontology.

[19] Airola E, Rasi P (2020). Domestication of a robotic medication-dispensing service among older people in Finnish Lapland. Hum Technol.

[20] Lindberg B, Nilsson C, Zotterman D, Söderberg S, Skär L (2013). Using information and communication technology in home care for communication between patients, family members, and healthcare professionals: a systematic review. Int J Telemed Appl.

[21] Lindberg T, Sandström B, Andersson EK, Borg C, Hjelm M, Nilsson L, Olsson A, Skär L (2021). Older persons' experience of eHealth services in home health care: a meta-ethnography eHealth services in home health care. Health Informatics J.

[22] Şahin E, Yavuz Veizi BG, Naharci MI (2024). Telemedicine interventions for older adults: a systematic review. J Telemed Telecare.

[23] Wang J, Fu Y, Lou V, Tan SY, Chui E (2021). A systematic review of factors influencing attitudes towards and intention to use the long-distance caregiving technologies for older adults. Int J Med Inform.

[24] Holthe T, Halvorsrud L, Lund A (2022). Digital assistive technology to support everyday living in community-dwelling older adults with mild cognitive impairment and dementia. Clin Interv Aging.

[25] Chelongar K, Ajami S (2021). Using active information and communication technology for elderly homecare services: a scoping review. Home Health Care Serv Q.

[26] Martínez-Alcalá CI, Pliego-Pastrana P, Rosales-Lagarde A, Lopez-Noguerola JS, Molina-Trinidad EM (2016). Information and communication technologies in the care of the elderly: systematic review of applications aimed at patients with dementia and caregivers. JMIR Rehabil Assist Technol.

[27] Bavngaard M, Lund A, Thordardottir B, Rasmussen E (2023). The uses and experiences of communication technology for home-dwelling older adults in a homecare services context: a qualitative systematic review. PROSPERO.

[28] Butler A, Hall H, Copnell B (2016). A guide to writing a qualitative systematic review protocol to enhance evidence-based practice in nursing and health care. Worldviews Evid Based Nurs.

[29] Coiera E (2006). Communication systems in healthcare. Clin Biochem Rev.

[30] Moser I (2019). Velferdsteknologi. En Ressursbok.

[31] Boyle LD, Husebo BS, Vislapuu M (2022). Promotors and barriers to the implementation and adoption of assistive technology and telecare for people with dementia and their caregivers: a systematic review of the literature. BMC Health Serv Res.

[32] EndNote. Clarivate.

[33] Ouzzani M, Hammady H, Fedorowicz Z, Elmagarmid A (2016). Rayyan-a web and mobile app for systematic reviews. Syst Rev.

[34] Polanin JR, Pigott TD, Espelage DL, Grotpeter JK (2019). Best practice guidelines for abstract screening large‐evidence systematic reviews and meta‐analyses. Res Synth Methods.

[35] Page MJ, McKenzie JE, Bossuyt PM, Boutron I, Hoffmann TC, Mulrow CD, Shamseer L, Tetzlaff JM, Akl EA, Brennan SE, Chou R, Glanville J, Grimshaw JM, Hróbjartsson A, Lalu MM, Li T, Loder EW, Mayo-Wilson E, McDonald S, McGuinness LA, Stewart LA, Thomas J, Tricco AC, Welch VA, Whiting P, Moher D (2021). The PRISMA 2020 statement: an updated guideline for reporting systematic reviews. BMJ.

[36] Atkins S, Lewin S, Smith H, Engel M, Fretheim A, Volmink J (2008). Conducting a meta-ethnography of qualitative literature: lessons learnt. BMC Med Res Methodol.

[37] CASP checklists. Critical Appraisal Skills Programme.

[38] Long HA, French DP, Brooks JM (2020). Optimising the value of the critical appraisal skills programme (CASP) tool for quality appraisal in qualitative evidence synthesis. Res Methods Med Health Sci.

[39] Correa G, Domènech M (2013). Care networking: a study of technical mediations in a home telecare service. Int J Environ Res Public Health.

[40] Criado TS, Domenech M (2015). Older people in a connected autonomy? Promises and challenges in the technologization of care. Span J Sociol Res.

[41] Gilbert AS, Croy S, Hwang K, LoGiudice D, Haralambous B (2021). Video remote interpreting for home-based cognitive assessments: stakeholders’ perspectives. Interpreting.

[42] Ilomäki S, Ruusuvuori J (2022). Preserving client autonomy when guiding medicine taking in telehomecare: a conversation analytic case study. Nurs Ethics.

[43] Karlsen C, Moe CE, Haraldstad K, Thygesen E (2019). Caring by telecare? A hermeneutic study of experiences among older adults and their family caregivers. J Clin Nurs.

[44] Lee K, Fields N, Cassidy J, Kusek V, Feinhals G, Calhoun M (2021). Caring callers: the impact of the telephone reassurance program on homebound older adults during COVID-19. Home Health Care Serv Q.

[45] Outila M, Kiuru H (2021). “Picturephone in my home”: actor-network theory and Foucauldian Discourse Analysis on Northern Finnish older adults starting to use a video conferencing service. J Technol Hum Serv.

[46] Read Paul L, Salmon C, Sinnarajah A, Spice R (2019). Web-based videoconferencing for rural palliative care consultation with elderly patients at home. Support Care Cancer.

[47] Schmidt S, Behrens J, Lautenschlaeger C, Gaertner B, Luderer C (2019). Experiences with combined personal-online case management and the self-reliance of older people with multimorbidity living alone in private households: results of an interpretative-hermeneutical analysis. Scand J Caring Sci.

[48] Similä H, Harjumaa M, Isomursu M, Ervasti M, Moilanen H (2014). Video communication in remote rehabilitation and occupational therapy groups. Phys Occup Ther Geriatr.

[49] Stokke R (2017). "Maybe we should talk about it anyway": a qualitative study of understanding expectations and use of an established technology innovation in caring practices. BMC Health Serv Res.

[50] Stokke R (2018). Older people negotiating independence and safety in everyday life using technology: qualitative study. J Med Internet Res.

[51] Åkerlind C, Martin L, Gustafsson C (2018). eHomecare and safety: the experiences of older patients and their relatives. Geriatr Nurs.

[52] Thomas J, Harden A (2008). Methods for the thematic synthesis of qualitative research in systematic reviews. BMC Med Res Methodol.

[53] Sandelowski M, Barroso J (2006). Handbook for Synthesizing Qualitative Research.

[54] Braun V, Clarke V (2021). Thematic Analysis: A Practical Guide.

[55] Pols J, Willems D (2011). Innovation and evaluation: taming and unleashing telecare technology. Sociol Health Illn.

[56] Malterud K (2019). Qualitative Metasynthesis: A Research Method for Medicine and Health Sciences.

[57] Silva RC, Zechner M, Yaylagul-Korkmaz N, Efthymiou A, Jøranson N (2022). Experienced barriers in the use of ICT for social interaction in older adults ageing in place: a qualitative systematic review protocol. PROSPERO.

[58] Hovland H, Thygesen E, Haraldstad K, Karlsen C (2022). Older adults’ experiences with digital technology to maintain social contact: a qualitative meta-synthesis protocol. PROSPERO.

[59] Thordardottir B, Malmgren Fänge A, Lethin C, Rodriguez Gatta D, Chiatti C (2019). Acceptance and use of innovative assistive technologies among people with cognitive impairment and their caregivers: a systematic review. Biomed Res Int.

[60] Olsson T, Viscovi D (2018). Warm experts for elderly users: who are they and what do they do?. Human Technol.

[61] Peng L, Man SS, Chan AH, Ng JY (2022). Personal, social and regulatory factors associated with telecare acceptance by Hong Kong older adults: an indication of governmental role in facilitating telecare adoption. Int J Hum Comput Interact.

[62] Martín-García AV, Redolat R, Pinazo-Hernandis S (2022). Factors influencing intention to technological use in older adults. The TAM model application. Res Aging.

[63] Cimperman M, Makovec Brenčič M, Trkman P (2016). Analyzing older users' home telehealth services acceptance behavior-applying an extended UTAUT model. Int J Med Inform.

[64] Johannessen LE, Nordtug M, Haldar M (2023). Multi-site domestication: taming technologies across multiple institutional settings. Inf Commun Soc.

[65] Hämäläinen A, Hirvonen H (2020). Electronic health records reshaping the socio-technical practices in long-term care of older persons. Technol Soc.

[66] Søraa RA, Nyvoll P, Tøndel G, Fosch-Villaronga E, Serrano JA (2021). The social dimension of domesticating technology: interactions between older adults, caregivers, and robots in the home. Technol Forecast Soc Change.

[67] Jalil S, Hardy D, Myers T, Atkinson I (2014). But it doesn't go with the décor: domesticating a telemedicine diabetes intervention in the home. Proceedings of the 26th Australian Computer-Human Interaction Conference on Designing Futures: The Future of Design.

[68] Saborowski M, Kollak I (2015). “How do you care for technology?” – Care professionals' experiences with assistive technology in care of the elderly. Technol Forecast Soc Change.

[69] Greenhalgh T, Wherton J, Papoutsi C, Lynch J, Hughes G, A'Court C, Hinder S, Fahy N, Procter R, Shaw S (2017). Beyond adoption: a new framework for theorizing and evaluating nonadoption, abandonment, and challenges to the scale-up, spread, and sustainability of health and care technologies. J Med Internet Res.

[70] Oudshoorn N (2011). Telecare Technologies and the Transformation of Healthcare.

[71] Pols J (2012). Care at a Distance: On the Closeness of Technology.

[72] Borg J, Gustafsson C, Landerdahl Stridsberg S, Zander V (2023). Implementation of welfare technology: a state-of-the-art review of knowledge gaps and research needs. Disabil Rehabil Assist Technol.

[73] Peine A, Neven L (2011). Social-structural lag revisited. Gerontechnology.

[74] Nordtug M, Assing Hvidt E, Lüchau EC, Grønning A (2022). General practitioners' experiences of professional uncertainties emerging from the introduction of video consultations in general practice: qualitative study. JMIR Form Res.

[75] Saini M, Shlonsky A (2012). Systematic Synthesis of Qualitative Research.

