# Supplementary material for: The Uses and Experiences of Synchronous Communication Technology for Home-Dwelling Older Adults in a Home Care Services Context: Qualitative Systematic Review
Source: J Med Internet Res. 2024 Nov 22;26:e59285. doi: 10.2196/59285 (PMC11624454; doi:10.2196/59285)
Supplement: Multimedia Appendix 3 [file jmir_v26i1e59285_app3.docx]

Studies excluded because of insufficient methodological quality.

| Excluded studies | Reasons for exclusion |
| --- | --- |
| Fiorini L, Sorrentino A, Pistolesi M, Becchimanzi C, Tosi F, Cavallo F. Living With a Telepresence Robot: Results From a Field-Trial. IEEE robotics and automation letters. 2022;7(2):5405-12. DOI: 10.1109/LRA.2022.3155237 | The study’s research design was not found to be consistent with the research aims stated and a question of experience was primarily answered by using quantitative measures. Qualitative results, that is, data obtained during the debriefing meetings with caregivers only take up a fragment of the results. The qualitative data collection is not described adequately, as information regarding the method of collection, process of collection, and the type of data collected is either vague or missing. Furthermore, the qualitative results present 6 categories, but the authors do not describe how they arrived at these 6 categories nor their process of analysis. Similarly, the qualitative findings are not presented in a transparent manner: there is no comment on the reporting format, no headings, and no quotes from participants. No comments are made regarding the theoretical underpinnings of the study despite its mixed-methods design. |
| Choi NG, Caamano J, Vences K, Marti CN, Kunik ME. Acceptability and effects of tele-delivered behavioral activation for depression in low-income homebound older adults: in their own words. Aging Ment Health. 2021;25(10):1803-10. DOI: 10.1080/13607863.2020.1783516 | Quantitative survey items were primarily used to explore the participant experience and acceptability of the technology. The research design does not seem immediately appropriate to address the aim of the study, as the data collection used for the study’s qualitative component seems incidental or, in the authors’ own words, “unsolicited.” The authors vary between describing the data collection setting as interviews and sessions of Tele-BA. Furthermore, the process of data collection is only minimally described, with no presentation of the interview guide and no description of the type of data. In addition, the authors mention collating categories with an eye for presentation, implying that the reason for collating the themes was not analytical. The original process of analysis for the qualitative data, if one exists, is indiscernible. |
